# Case of Partial Paralysis in Children

**Published:** 1840-07

**Authors:** 


### CASES OF PARTIAL PARALYSIS IN CHILDREN.

In the month of June when in Xenia, Ohio, Dr. Templeton afforded us an opportunity of seeing five children affected with partial paralysis. The resemblance to each other, of these cases, and the *simultaneous* occurrence of four of them, induced us to record the following particulars.

*Case* 1.—Mr. Sheppard's son, aged 15 months, generally enjoyed good health, and could walk very well, when a little more than a year old. About the middle of May, he became costive and fretful, and after a few days had decided fever. Three days after the supervention of the latter, palsy of the lower extremeties came on. It was perfect, that is to say, all power of the will over the muscles seemed to be gone, but the sensibility of the skin remained. The arms were unaffected, but the muscles of the trunk of the body were enfeebled, so that he could not sit up. The fever soon ceased. He took calomel and other cathartics. When we saw him, June 30, the right leg was nearly restored—the left remaining almost motionless. His general health appeared perfect. He had no spinal tenderness.

*Case* 2.—Mr. Moore's daughter, aged 20 months, was healthy from her birth, and walked when nine months old. In the latter part of May, she became costive, feverish, and was observed to have a weak and husky voice. She now and then screamed out in sleep, and at the end of a week had one spell of vomiting. Suddenly she lost the power of motion in her lower extremities, which for two days and nights were very cold. Their sensibility remained unimpaired. For three weeks, she could not even crawl nor stand; she then began to use the right limb moderately, and at the time we saw her, the left was moved in a slight degree, when the sole of the foot was tickled. An examination of the spine disclosed nothing. Before the paralysis supervened, a few drops of cathartic medicine were administered.

*Case* 3.—The son of Mr. Charters, 5 years old, was generally in good health, and properly developed both in body and mind, till about the middle of May last, when he was observed to be costive, and complained of headache and sick stomach. A cathartic was administered and he got better; but in a week he was again costive and feverish, with a dry husky cough. Cathartics were again exhibited and were found to operate very tardily. In a few days it was observed, that when placed on his feet his legs were nearly paralysed, and he seemed to experience pain. Worm medicines, and calomel as a cathartic, were administered freely. No worms were discharged. In a few days he made efforts to walk, when assisted, but turned his toes outward, and dragged his feet. When we saw him, June 30, this debility and dragging still continued, but he was much better. It should be stated, that previously to the attack, his appetite was voracious and that he ate a great deal of meat.

*Case* 4.—A daughter of the same parents, aged 10 months, had been generally in good health up to the middle of May, when she became costive, irritable and feverish. The gums of the middle upper teeth, in front, being tumid and red, were cut. In a few days she partially lost the use of all her limbs, and the movements of her head were greatly weakened. Her voice was feeble and husky, and she had a short, dry, hoarse cough. The debility of her limbs increased; but in a day or two began to abate in all but the left leg and arm, which became nearly motionless. At the time of our examination, she had begun to move her limbs on that side a little—chiefly however by the flexor muscles, the extensors seeming to be entirely paralysed. Dr. Templeton could detect no spinal tenderness. She was treated with cathartic medicines, and was manifestly convalescent when we saw her.

*Case* 5.—Another, 3 years old, was shown to us, who in infancy was healthy, but when she began to use her hands and feet, it was observed that those of the left side were exceedingly inactive and feeble, compared with the others. When 20 months old, she began to walk, but still does it imperfectly—presenting her right side and dragging her left foot, with the toes turned a little out. Her fingers of the left hand are perpetually flexed. She often opens them with her right hand, so as to grasp what she would hold. She cannot protrude her tongue beyond her lower lip. The saliva drivels from her mouth, and in masticating, a portion of the food escapes from its corners, particularly the left, but she has no retraction of the right. She articulates very few words. Her mind is shrewd—her observations acute—her temper irritable—her communication with those around in a great degree pantomimic.

She was never subjected to any medical treatment. The mother of this child had a brother affected with epilepsy. No other nervous disease in the family.

The occurrence of three of these cases, so much resembling each other in the family of Charters, would seem to indicate a family predisposition; while the occurrence of two cases in his family, one in Shephard's and one in Moore's, nearly at the same time, the whole in one street and within a few squares of one another, would appear equally to indicate a common cause. What that cause might have been, is not known. Dr. Templeton, at first suspected that the children of Charters had been injured with lead, as he worked more or less in paints, and as the constipation, and the paralysis of the extensor muscles, appeared characteristic of the impress of that metal. When two other children, however, in two neighboring houses, were found to labor under the same affection, that theory was relinquished. D.

